# A Case of Giant Cell Arteritis Presenting After COVID-19 Vaccination: Is It Just a Coincidence?

**DOI:** 10.7759/cureus.21608

**Published:** 2022-01-25

**Authors:** Christopher S Greb, Zineb Aouhab, Daniel Sisbarro, Elnaz Panah

**Affiliations:** 1 Internal Medicine, Loyola University Medical Center, Maywood, USA; 2 Rheumatology, Loyola University Medical Center, Maywood, USA; 3 Pathology, Loyola University Medical Center, Maywood, USA

**Keywords:** vaccine, immunization, covid, covid-19, covid 19, temporal artertitis, gca, giant cell arteritis

## Abstract

Giant cell arteritis (GCA) is a large vessel vasculitis with variable presentations, including fevers, myalgias, headache, and jaw claudication. A particularly concerning symptom is transient vision loss, which may become irreversible without prompt recognition and treatment. The pathogenesis of GCA is incompletely understood, but it seems that the innate and adaptive immune systems play a key role in vessel inflammation, remodeling, and occlusion. We present a case of a 79-year-old male who developed GCA two days after he received his second dose of a COVID-19 mRNA vaccine. He presented with headaches, fever, and myalgias. Lab workup revealed elevated inflammatory markers, with C-reactive protein (CRP) 272 mg/L (<8.1 mg/L) and erythrocyte sedimentation rate (ESR) 97 mm/hr (0-20mm/hr). Imaging of the head, with CT and MRI, was unremarkable. His headache persisted despite supportive treatment, and he developed new, transient blurred vision, which increased suspicion for GCA. He underwent bilateral temporal artery biopsies, which were consistent with GCA. His symptoms resolved quickly with oral prednisone 60mg daily, and his inflammatory markers returned to normal within a month. A review of the literature revealed several case reports of giant cell arteritis following influenza vaccination. However, no large-scale studies have demonstrated a causal relationship between GCA and immunization. Our case demonstrates the first instance of GCA following a COVID-19 mRNA vaccine. We propose that the upregulated immune response to the vaccine acted as a trigger for GCA in this patient with predisposing factors. While causation cannot be determined based on one case alone, our case demonstrates an opportunity for further research into the relationship between vasculitis and immunizations. Despite this isolated case, the proven benefits of COVID-19 mRNA vaccines significantly outweigh any theoretical risk of immune dysregulation following administration.

## Introduction

Giant cell arteritis (GCA) is a rare, large-vessel vasculitis that affects the aorta and its branches [1]. It has variable presentations, including fever, myalgias, headache, jaw claudication, and transient vision loss. It must be recognized promptly to prevent serious consequences, most notably irreversible vision loss [2]. This can prove difficult, as the triggering events and pathogenesis of GCA are incompletely understood. However, it seems that the innate and adaptive immune systems play a significant role in the amplification of inflammatory pathways that lead to vessel inflammation, remodeling, and occlusion [3]. Several pathogens have been proposed as potential triggers for giant cell arteritis due to their identification in GCA temporal artery biopsies, such as *chlamydia pneumonia*, parvovirus B19, human herpes viruses, human papillomavirus, and *Burkholderia*-like bacterium. However, no microorganism or viral pathogen has demonstrated a definitive causal relationship with GCA [4]. In the innate immune system, toll-like receptors activate dendritic cells, which activate T cells that infiltrate large and medium-sized blood vessels. Activated T cells are regulated by immune checkpoints, such as programmed death-ligand 1 (PD-L1), which may be ineffective in GCA [3]. This idea is supported by the development of giant cell arteritis after checkpoint inhibitor immunotherapy, such as ipilimumab, in some patients with malignant melanoma [3]. In the adaptive immune system, increased T cell production of pro-inflammatory cytokines, such as IL-17A, and decreased activity of regulatory T cells may contribute to the excessive vessel inflammation in giant cell arteritis [5].

## Case presentation

**Figure 1:**
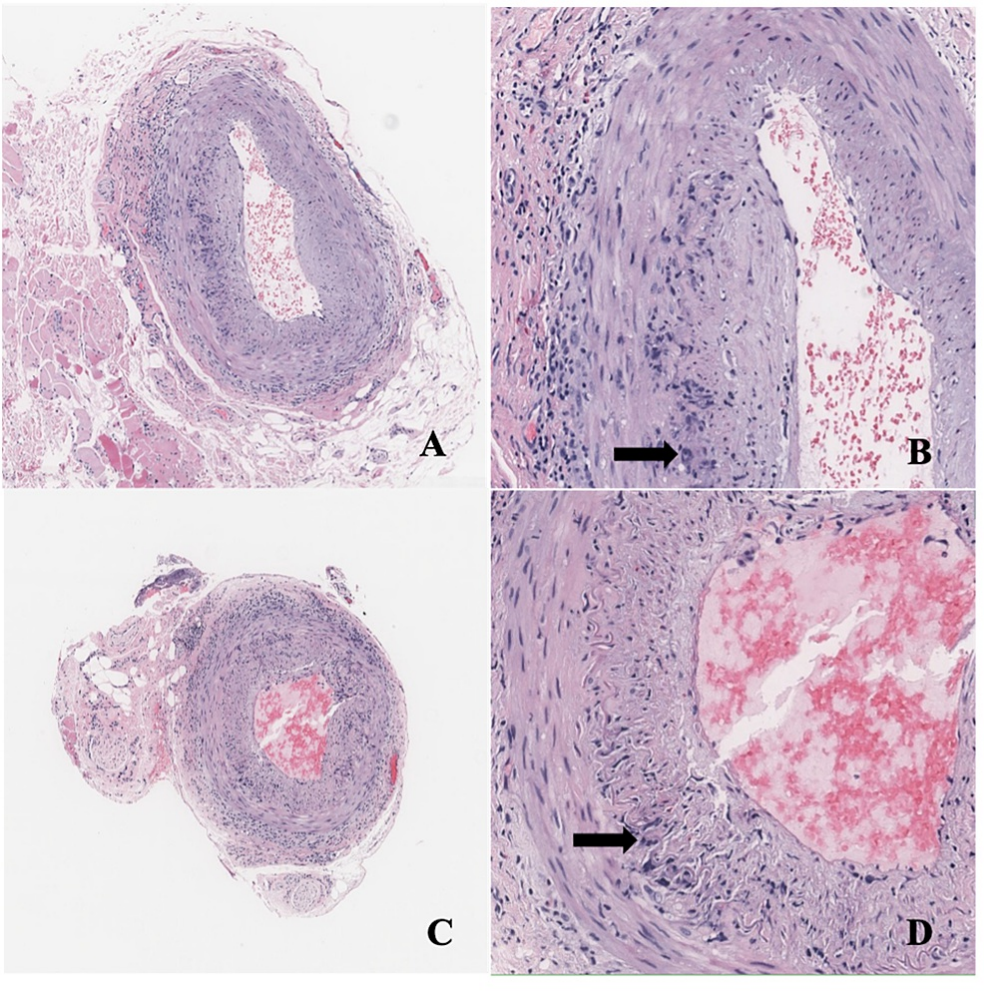
Temporal Artery Biopsies Right temporal artery (A, B) and left temporal artery (C, D): Medium size muscular artery with intramural inflammatory infiltrates composed of histiocytes, lymphocytes, and eosinophils. Multinucleated giant cells can be identified (arrows). The infiltrate is concentrated at the level of internal elastic lamina and adventitia. Hematoxylin and eosin (H&E) stain, magnification 50x (A, C), 200x (B, D).

We describe a case of a 79-year-old man who presented with nightly headaches for three weeks and transient blurry vision for one week. He reported sharp, debilitating frontal headaches that radiated over the temples bilaterally. The headaches were associated with fever, fatigue, and myalgias. The patient’s past medical history included hypertension, hyperlipidemia, atrial fibrillation, hypothyroidism, prostate cancer, and rectal cancer. Symptoms began two days after he received the second dose of a COVID-19 mRNA vaccine. He was seen at an outside hospital emergency department at the onset of symptoms. Infectious workup with chest radiography and urinalysis was unremarkable. He was diagnosed with a mild vaccine reaction and discharged from the emergency department. His symptoms persisted for another week, and he presented to our institution, where he was admitted for further evaluation. Lab workup was remarkable for leukocytosis to 16.9 K/uL (3.5-10.5K/uL), mild transaminitis, and elevated inflammatory markers, with CRP 272 mg/L (<8.1 mg/L) and ESR 97 mm/hr (0-20 mm). Infectious workup, with urinalysis and blood cultures, and head imaging, with CT and MRI, were unremarkable. His headache improved slightly with acetaminophen and IV fluids, and he was discharged after three days. At his follow-up office visit a week later, his headache had become much worse with associated fatigue, scalp tenderness, and three episodes of transient blurred vision in the right and left eye. Each episode resolved after five minutes. He was readmitted due to concern for GCA. On admission, his ophthalmic exam was unremarkable, but he was treated with 60mg oral prednisone due to high suspicion for GCA. His headache completely resolved less than 12 hours after the administration of steroids. Bilateral temporal artery biopsies were performed the next day, which can be seen in Figures 1A-1D. 

**Figure 2:**
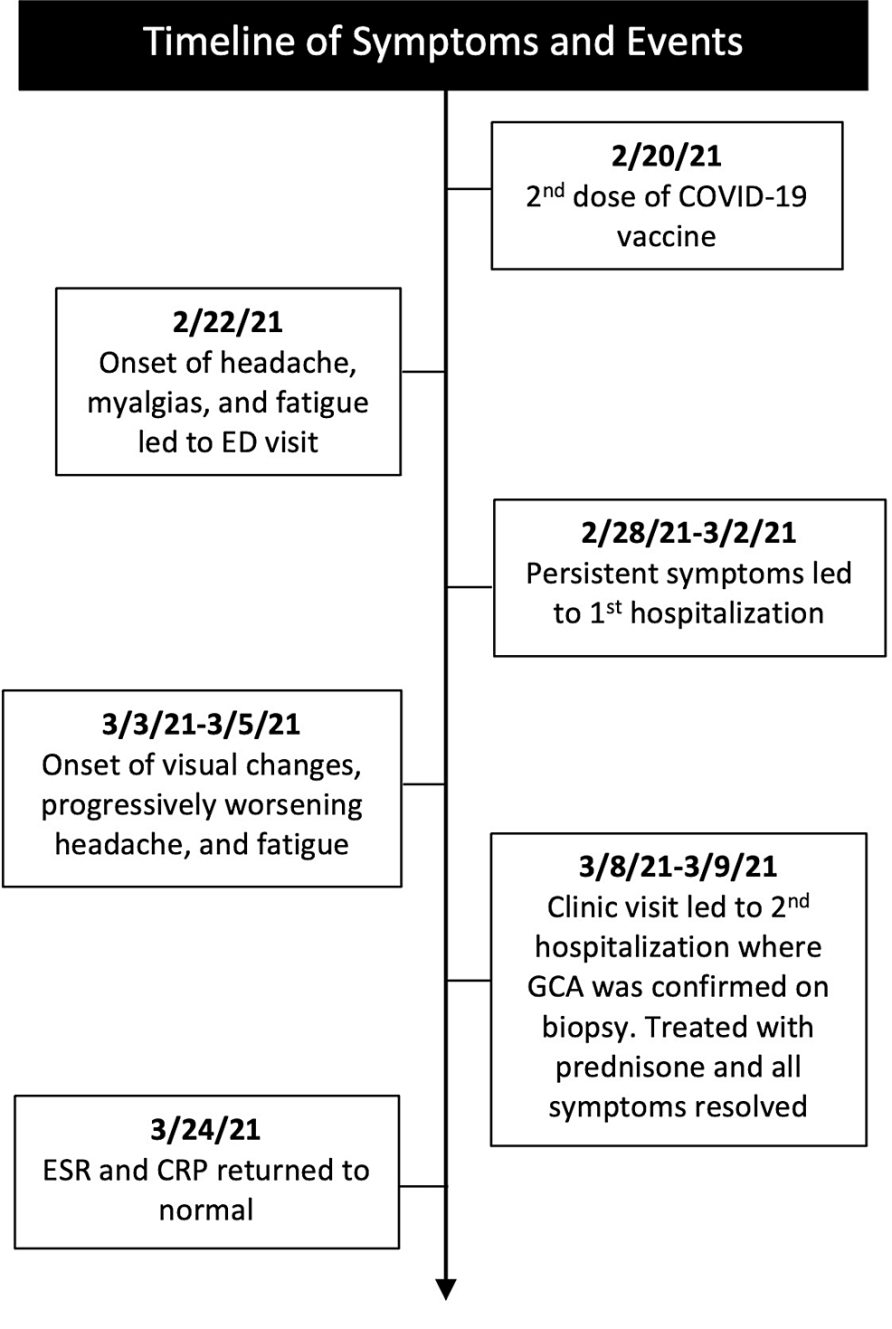
Timeline of Symptoms and Events

Both biopsies revealed patchy intramural inflammatory infiltrates composed of lymphocytes and rare multinucleated giant cells at the internal lamina and adventitia consistent with a diagnosis of GCA. The patient was discharged on high-dose prednisone (60mg daily), and three weeks later, his CRP and ESR improved to <1 mg/L and 2 mm/hr, respectively. He was tapered to prednisone 50mg daily by his six-week follow-up appointment without recurrence of symptoms. The timeline of the patient’s symptoms and healthcare interactions is illustrated in Figure 2.

## Discussion

Concerns have been raised about the relationship between vaccinations and autoimmune disease, including vasculitis. A special focus has been placed on molecular mimicry and the role of adjuvants [6]. These adjuvants increase the innate immune response to foreign antigens, which cause concerns that they may induce reactivity to self-antigens [7]. The COVID-19 mRNA vaccine administered to the patient, in this case, did not contain traditional adjuvants, such as aluminum, seen in other non-mRNA vaccines. However, RNA has intrinsic adjuvant activity, which induces a strong innate immune response [8]. A review of the literature by Guiamares et al. in 2015 revealed 15 cases of GCA following influenza vaccination [9]. Case reports of varying autoimmune diseases after vaccination led Schoenfeld et al. to coin the term autoimmune/inflammatory syndrome induced by adjuvants (ASIA syndrome) to describe a spectrum of immune dysregulation following vaccination [10]. Although a small number of case reports have been published, no epidemiologic studies have shown a definitive increase in autoimmune disease following vaccination. A committee convened in 2015 by the International Life Sciences Institute (ILSI) and Health and Environmental Sciences Institute (HESI) conducted an extensive literature review and concluded that there is no compelling evidence supporting the association of vaccine adjuvants and autoimmunity [11]. We reviewed the PubMed and Medline databases by searching case reports with the term “giant cell arteritis” combined with the name of several commonly administered vaccines. No date restriction was placed on the results. The number of cases associated with each vaccine is shown in Table 1.

**Table 1:** Reported Cases of Giant Cell Arteritis Following Vaccination

Vaccine	GCA Cases	References
Influenza	24	[11-19]
Varicella Zoster	1	[20]
COVID-19	0	-

The review revealed 24 documented cases of GCA after influenza vaccination and one case after varicella-zoster vaccination. No cases of giant cell arteritis have been reported following hepatitis A, hepatitis B, tetanus, measles, mumps, rubella, pneumococcal, meningitis, or COVID-19 vaccinations.

## Conclusions

Our case represents the first reported case of giant cell arteritis following vaccination with a COVID-19 mRNA vaccine. We propose that the upregulated immune response to the vaccine acted as a trigger for GCA in this patient with predisposing risk factors. However, causation cannot be reliably determined based solely on the timing of symptoms in this isolated case. Our case demonstrates the opportunity for further large-scale studies examining the incidence of GCA in treatment and control groups to better characterize the relationship between GCA and vaccination. Currently, data is limited regarding this relationship. The poor understanding of GCA inciting factors in the literature means clinicians must be diligent when looking for sources of immune hyperreactivity in patients presenting with symptoms suggestive of GCA. While our case suggests opportunities for further research, we strongly endorse continued COVID-19 vaccination, even in elderly patients and those of Northern European ancestry who may be predisposed to developing GCA. During a global pandemic with the highly infectious and lethal COVID-19 virus, the benefits of vaccination far outweigh any theoretical risk of immune dysregulation following administration.
